# Supplementary material for: Safety and Effectiveness of Perioperative Hyperthermic Intraperitoneal Chemotherapy with Gemcitabine in Patients with Resected Pancreatic Ductal Adenocarcinoma: Clinical Trial EudraCT 2016-004298-41
Source: Cancers (Basel). 2024 Apr 28;16(9):1718. doi: 10.3390/cancers16091718 (PMC11083892; doi:10.3390/cancers16091718)
Supplement: Supplementary file 1 [file cancers-16-01718-s001.zip › cancers-2912718-supplementary.pdf]

Characteristics		**Group II** (n=21)	**Group I** (n=21)	***P*-value**
**Age (years, *M ± SEM*)**		64 ± 1.7	69.3 ± 2	0.024
**Overall survival (months, *median*)**		17.1 (0.6-61.9)	18 (0.8-61.8)	ns
**Disease-free survival (months, *median*)**		14 (2-66)	10 (2-66)	ns
**Operative time (minutes, *median*)**		350 (280-420)	300 (120-360)	0.002
**Hospital stay (days, *median*)**		11 (7-28)	17 (4-69)	ns
**PaCSCs (CXCR4+CD133+EpCAM)**		19.7 ± 8.2	13.8 ± 7.1	ns
**Sex**		***n* (%)**	***n* (%)**	
	Male	6 (29)	13 (62)	0.03
	Female	15 (71)	8 (38)	
**Symptoms**				
	HTA	10 (48)	9 (43)	ns
	DM	9 (43)	7 (33)	ns
	DL	10 (48)	6 (29)	ns
	Jaundice	17 (81)	12 (57)	ns
	Abdomnal pain	8 (38)	7 (33)	ns
	Constitutional syndrome	5 (24)	5 (24)	ns
**Resections**				
	TP without splenectomy	1 (5)	4 (19)	ns
	TP with splenectomy	9 (43)	5 (24)	ns
	CDP	9 (43)	8 (38)	ns
	STP	1 (5)	1 (5)	ns
	DP	0	1 (5)	ns
	CCP	1 (5)	2 (10)	ns
**Pancreatitis complications**				
	Delayed gastric emptying	2 (10)	2 (10)	ns
	Pancreatic fistula (B, C)	0	1 (5)	ns
	Haemorrhage	0	2 (10)	ns
**Clavien-Dindo**				
	I	4 (19)	3 (14)	
	II	5 (24)	3 (14)	
	III	2 (10)	3 (14)	ns
	IV	0	1 (5)	
	V	1 (5)	1 (5)	
**Differentiation**				
	Good	4 (19)	3 (14)	
	Moderate	7 (33)	14 (67)	ns
	Poor	10 (48)	4 (19)	
**Invasion**				
	Neurologic invasion	15 (71)	15 (71)	ns
	Vascular invasion	7 (33)	8 (38)	ns
	Lymphatic Invasion	7 (33)	11 (52)	ns

**TNM**				
	Ia	2 (10)	5 (24)	
	Ib	6 (29)	3 (14)	
	IIa	1 (5)	2 (10)	ns
	IIb	6 (29)	8 (38)	
	III	6 (29)	3 (14)	
**Locoregional recurrence**		2 (10)	11 (52)	0.004
**Distant recurrence**		9 (43)	8 (38)	ns
**Mortality (> 30 days)**		13 (62)	13 (62)	ns

Parameters	Group II (n=21)	Group I (n=21)	*P*-value
Pre-Creatinina	0.9 ± 0.3	0.9 ± 0.1	ns
Creatinine 24h	4.1 ± 3.1	1.3 ± 0.1	0.034
Creatinina 7 days	1.0 ± 0.2	0.9 ± 0.2	ns
Pre-Urea	31.8 ± 2.0	39.5 ± 3.2	0.048
Urea 24h	32.2 ± 2.2	45.5 ± 5.9	ns
Urea 7 days	35.4 ± 4.6	43.7 ± 5.2	ns
Pre-BB	9.7 ± 4.0	6.6 ± 1.9	ns
BB 24h	3.9 ± 1.2	2.6 ± 0.6	ns
BB 7 days	3.0 ± 1.3	2.4 ± 0.7	ns
Pre-GOT	112.7 ± 33.9	125.8 ± 34.5	ns
GOT 24h	115.4 ± 26.2	127.3 ± 25.9	ns
GOT 7 days	45.3 ± 4.5	52.6 ± 14.8	ns
Pre-GPT	112.9 ± 27.7	169.5 ± 47.1	ns
GPT 24h	119.7 ± 21.8	120.0 ± 18.5	ns
GPT 7 days	51.3 ± 4.0	73.1 ± 18.5	ns
Pre-Glu	154.0 ± 18.8	144.9 ± 14.0	ns
Glu 24h	152.7 ± 11.6	140.0 ± 9.4	ns
Glu 7 days	178.7 ± 22.1	202.7 ± 29.9	ns
Pre-Na	196.5 ± 57.3	138.8 ± 1.0	ns
Na 24h	201.9 ± 61.8	140.2 ± 0.9	ns
Na 7 days	216.7 ± 79.5	138.2 ± 1.5	ns
Pre-K	10.7 ± 3.5	4.4 ± 0.1	ns
K 24h	5.4 ± 1.4	4.3 ± 0.1	ns
K 7 days	6.6 ± 2.6	4.1 ± 0.1	ns
Pre-Cl	146.4 ± 43.2	102.3 ± 1.0	ns
Cl 24h	159.9 ± 51.0	106.4 ± 0.9	0.046
Cl 7 days	99.9 ± 1.6	100.0 ± 1.3	ns
Pre-Proact	91.6 ± 1.9	85.8 ± 3.1	ns
Proact 24h	77.0 ± 2.4	76.3 ± 3.2	ns
Proact 7 days	90.6 ± 2.5	87.2 ± 3.1	ns
Pre-PT	18.4 ± 6.6	13.3 ± 1.2	ns
TP 24h	32.0 ± 13.4	13.7 ± 0.9	ns

PT 7 days	30.2 ± 18.0	13.3 ± 1.0	ns
Pre-RBC	5.5 ± 1.3	4.1 ± 0.2	ns
RBC 24h	4.3 ± 0.9	3.4 ± 0.1	ns
RBC 7 days	5.3 ± 1.9	3.2 ± 0.1	ns
Pre-Pla	284.7 ± 17.6	282.0 ± 24.5	ns
Pla 24h	229.5 ± 18.3	228.5 ± 19.4	ns
Pla 7 days	293.8 ± 28.2	302.7 ± 41.5	ns
Pre-Le	18.9 ± 8.1	10.8 ± 1.1	ns
Le 24h	23.4 ± 9.6	14.8 ± 1.1	ns
Le 7 days	46.7 ± 31.6	13.8 ± 1.7	ns
Pre-Hb	22.0 ± 6.6	12.3 ± 0.4	ns
Hb 24h	13.7 ± 3.4	10.4 ± 0.4	ns
Hb 7 days	15.8 ± 5.8	9.7 ± 0.4	ns
Pre-Hct	66.4 ± 20.1	37.4 ± 1.3	ns
Hct 24h	40.2 ± 9.8	31.2 ± 1.2	ns
Hct 7 days	30.3 ± 1.0	29.4 ± 1.3	ns
Pre-PO_2	111.5 ± 47.6	122.6 ± 17.1	ns
PO_2 24h	135.7 ± 12.4	127.2 ± 6.7	ns
PO_2 7 days	100.2 ± 8.7	90.7 ± 14.4	ns
Pre-PCO_2	42.0 ± 2.4	40.6 ± 1.7	ns
PCO_2 24h	37.5 ± 2.4	41.7 ± 1.6	0.024
PCO_2 7 days	41.6 ± 2.5	48.8 ± 3.7	ns
Pre-pH	7.4 ± 0.1	7.3 ± 0.3	ns
pH 24h	9,0 ± 1.6	7.4 ± 0.1	ns
pH 7 days	7.4 ± 0.5	7.4 ± 0.2	ns
Pre-HCO_3	25.9 ± 1.8	22.7 ± 0.6	ns
HCO_3 24h	32.9 ± 11.3	26.2 ± 0.9	0.002
HCO_3 7 days	25.6 ± 1.5	34.2 ± 1.7	0.001
Pre-Lactic acid	9.8 ± 1.8	14.4 ± 2.9	ns
Lactic acid 24h	22.7 ± 4.1	18.7 ± 4.3	ns
Lactic acid 7 days	11.2 ± 2.3	11.8 ± 2.2	ns
